# Draft Genome Sequences of Six *Listeria monocytogenes* Strains Isolated from Dairy Products from a Processing Plant in Southern Italy

**DOI:** 10.1128/genomeA.00282-14

**Published:** 2014-04-10

**Authors:** Matteo Chiara, Anna Maria D’Erchia, Caterina Manzari, Alice Minotto, Cosimo Montagna, Nicoletta Addante, Gianfranco Santagada, Laura Latorre, Graziano Pesole, David S. Horner, Antonio Parisi

**Affiliations:** a Dipartimento di Bioscienze, Università degli Studi di Milano, Milano, Italy; b Dipartimento di Bioscienze, Biotecnologie e Biofarmaceutica, Università degli Studi di Bari Aldo Moro, Bari, Italy; c Istituto Zooprofilattico Sperimentale della Puglia e della Basilicata, Putignano, Italy; d Istituto Zooprofilattico Sperimentale della Puglia e della Basilicata, Matera, Italy; e Istituto di Biomembrane e Bioenergetica, Consiglio Nazionale delle Ricerche, Bari, Italy

## Abstract

Here we announce the draft genome sequences of 6 *Listeria monocytogenes* strains from ricotta cheese produced in a dairy processing plant located in southern Italy and potentially involved in a multistate outbreak of listeriosis in the United States.

## GENOME ANNOUNCEMENT

*Listeria monocytogenes* is a Gram-positive opportunistic pathogen widely associated with outbreaks of food poisoning (1). A 2012 multistate outbreak of listeriosis in the United States was epidemiologically associated with an Italian cheese product. The microbiological investigations performed on cheese lots recalled from the involved facility allowed the identification of 71 different contaminated batches. In total, 430 *L. monocytogenes* isolates were genotyped using a multilocus variable-number tandem-repeat analysis (MLVA) protocol to assess their identity, and 6 distinct profiles (numbered from I to VI) were identified. Up to 10 isolates for each MLVA profile were also subjected to multilocus sequence typing (MLST) as previously described (2). All the tested isolates of the same MLVA type shared their sequence types (ST), although MLVA type I and VI were both of ST8. One strain for each MLVA type (Lm_1823 [ST8], Lm_1824 [ST2], Lm_1840 [ST101], Lm_1880 [ST121], Lm_1886 [ST5], and Lm_1889 [ST8]) was subjected to whole-genome shotgun sequencing.

Genomic DNA was extracted using the QIAmp DNA minikit (Qiagen, Milan, Italy), according to the manufacturer’s protocol. The library was prepared from the extracted genomic DNA using the Nextera XT DNA sample preparation kit (Illumina, San Diego, CA) and a 2× 250-nucleotide (nt) paired-end sequencing run was performed on an Illumina MiSeq platform. The reads were trimmed and *de novo* assembled using the Velvet software (3). The resulting assemblies have >135× coverage and generated 31, 28, 19, 27, 16, and 24 contigs representing the genomes of *L. monocytogenes* strains Lm_1886, Lm_1889, Lm_1823, Lm_1824, Lm_1840, and Lm_1880, respectively. The estimated genome size is 2.9 Mb, with a G+C content of 37.5% for each strain. Genome annotation, performed using the Rapid Annotations using Subsystems Technology (RAST) server (4), suggested that the genomes contain 2,944, 3,004, 3,053, 2,969, 3,003, and 3,027 protein-coding genes for strains Lm_1886, Lm_1889, Lm_1823, Lm_1824, Lm_1840, and Lm_1880, respectively. The availability of these *L. monocytogenes* genome sequences will facilitate in-depth studies of these isolates and their potential association with the American listeriosis outbreak and will also facilitate the development of new, more comprehensive genotyping assays for *L. monocytogenes* strains.

### Nucleotide sequence accession numbers.

The draft genome sequences described here have been deposited at DDBJ/EMBL/GenBank under the accession numbers AZIX00000000, AZIY00000000, AZIU00000000, AZIV00000000, AZIW00000000, and AZIZ00000000 for isolates Lm_1886, Lm_1889, Lm_1823, Lm_1824, Lm_1840, and Lm_1880, respectively.
